# HCN1 Channels Enhance Rod System Responsivity in the Retina under Conditions of Light Exposure

**DOI:** 10.1371/journal.pone.0147728

**Published:** 2016-01-25

**Authors:** Vithiyanjali Sothilingam, Stylianos Michalakis, Marina Garcia Garrido, Martin Biel, Naoyuki Tanimoto, Mathias W. Seeliger

**Affiliations:** 1 Division of Ocular Neurodegeneration, Institute for Ophthalmic Research, Centre for Ophthalmology, Eberhard Karls University of Tübingen, Schleichstr. 4/3, D-72076, Tübingen, Germany; 2 Center for Integrated Protein Science Munich CIPSM, Department of Pharmacy-Center for Drug Research, Ludwig-Maximilians-Universität München, Butenandtstr. 5–13, D-81377, München, Germany; University of Florida, UNITED STATES

## Abstract

**Purpose:**

Vision originates in rods and cones at the outer retina. Already at these early stages, diverse processing schemes shape and enhance image information to permit perception over a wide range of lighting conditions. In this work, we address the role of hyperpolarization-activated and cyclic nucleotide-gated channels 1 (HCN1) in rod photoreceptors for the enhancement of rod system responsivity under conditions of light exposure.

**Methods:**

To isolate HCN1 channel actions in rod system responses, we generated double mutant mice by crossbreeding *Hcn1*^-/-^ mice with *Cnga3*^-/-^ mice in which cones are non-functional. Retinal function in the resulting *Hcn1*^-/-^ *Cnga3*^-/-^ animals was followed by means of electroretinography (ERG) up to the age of four month. Retinal imaging via scanning laser ophthalmoscopy (SLO) and optical coherence tomography (OCT) was also performed to exclude potential morphological alterations.

**Results:**

This study on *Hcn1*^-/-^ *Cnga3*^-/-^ mutant mice complements our previous work on HCN1 channel function in the retina. We show here in a functional rod-only setting that rod responses following bright light exposure terminate without the counteraction of HCN channels much later than normal. The resulting sustained signal elevation does saturate the retinal network due to an intensity-dependent reduction in the dynamic range. In addition, the lack of rapid adaptational feedback modulation of rod photoreceptor output via HCN1 in this double mutant limits the ability to follow repetitive (flicker) stimuli, particularly under mesopic conditions.

**Conclusions:**

This work corroborates the hypothesis that, in the absence of HCN1-mediated feedback, the amplitude of rod signals remains at high levels for a prolonged period of time, leading to saturation of the retinal pathways. Our results demonstrate the importance of HCN1 channels for regular vision.

## Introduction

The retina is able to respond to a wide range of light stimuli by the use of the two photoreceptor subtypes, rods and cones, whose signals converge at different sites in the retina. The highly sensitive rods are able to detect already very low amounts of light, whereas the less sensitive cones mainly contribute to perception under brighter light levels. At intermediate (mesopic) conditions, there is a considerable overlap of the two systems where both photoreceptor types contact onto the same downstream neurons [1, 2]. In order to facilitate a seamless, light-dependent transition from rod- to cone-dominated retinal signalling, their dynamic range has to be tightly controlled.

In the retina, the wide response range is implemented not only at the level of photoreceptors, but also downstream via the use of parallel, independent pathways and intensity-dependent convergence of signals at each stage of retinal processing [3–5]. The primary rod pathway operates at dim light conditions and connects rods via rod bipolar cells to AII amacrine cells and eventually the cone ON pathway. It also suppresses cone OFF signalling. The secondary rod pathway becomes operational at mesopic illuminations. Rod signals access the cone ON pathway via rod-cone electrical coupling through gap junctions [6–9]. In addition, horizontal connections at each level of signal processing (e.g. via gap junctions) are important for several aspects of vision [10].

The dynamic range of rod vision is further regulated by a number of feedback mechanisms. Here, we explored the actions of Hyperpolarization-activated and cyclic nucleotide-gated 1 (HCN1) channels in rods. HCN1 channels are strongly expressed in photoreceptor inner segments [11–13] and are activated after a short delay when light exposure closes CNG channels in the outer retina and hyperpolarizes the membrane potential of photoreceptors. Increased opening of HCN1 channels causes an inward current that drives the membrane potential back to the resting state [13–16]. In previous investigations, we have focused particularly on the importance of HCN1 channels for proper rod-cone-interaction [16]. Here, we studied the isolated rod-specific actions via a genetic impairment of cone function, as usually the intrusion of cone system contributions at higher light intensity renders a rod-specific analysis difficult [15, 16]. For this purpose, we crossbred *Hcn1*^-/-^ mice [17] with *Cnga3*^-/-^ mice [18] in order to obtain *Cnga3*^-/-^ *Hcn1*^-/-^ double knockout mice (DKO) with abolished cone function. The resulting DKOs were examined functionally by means of *in vivo* electrophysiology (ERG) using single flash and flicker paradigms under scotopic and mesopic conditions. *In vivo* retinal imaging, using optical coherence tomography (OCT) and scanning laser ophthalmoscopy (SLO), was also performed to exclude potential morphological alterations.

## Materials and Methods

### Ethics Statement

All the experimental procedures regarding animals were performed according to the ARVO Statement for the Use of Animals in Ophthalmic and Vision Research and the law of animal experimentation issued by the German Government, and were finally approved by the local authorities (Regierungspraesidium Tuebingen).

### Animals

Two mouse lines were used in this study: the *Cnga3*^-/-^ single mutant [18] and *Cnga3*^-/-^ *Hcn1*^-/-^ double knockout line (DKO). DKOs were generated by crossbreeding single *Hcn1*^-/-^ and *Cnga3*^-/-^ animals and subsequent breeding of F1 heterozygotes. We identified double mutant mice in the F2 generation by PCR analysis of genomic DNA as described for the individual lines [17, 18]. All mice were examined at the age of 1 month because firstly retinal development is usually complete at this time point. Secondly, this time point is optimal for the analysis of primary functional changes due to a genetic defect in mice in-dependent of secondary changes (e.g degeneration) [19–21]. Lack of cone photoreceptor function due to a genetic ablation of the *Cnga3* gene has been found to secondarily induce synaptic remodelling to some degree [22]. Nevertheless, a functional alteration of rod system activity has so far not been described and was also not observed in previous studies involving ERG examinations [19–21]. So, this aspect remains at present of a theoretical nature.

### Electroretinography (ERG)

ERGs were performed according to procedures described previously [19, 20] using a Ganzfeld bowl, a signal amplifier, and a PC-based control and recording unit (Toennies Multiliner Vision, Viasys Healthcare, Höchberg, Germany). Custom-made gold wire rings and stainless steel needles (SEI EMG, Cittadella, Italy) were used as active or reference and ground electrodes, respectively. ERGs were recorded binocularly from the corneal surface. Mice were dark-adapted over night. For anaesthesia, a combination of ketamine (66.7 mg per kg body weight) and xylazine (11.7 mg per kg body weight) was utilized. Pupils were dilated prior to the experiments (Mydriaticum Stulln, Stulln, Germany).

#### Single-flash ERG

Single flash responses were obtained under dark-adapted conditions (without any background illumination). Stimulus intensity from white flashes were categorized into low scotopic (0.1 and 1 mcd*s/m^2^), high scotopic (10 mcd*s/m^2^), low mesopic (0.03–0.3 cd*s/m^2^) and high mesopic (1–25 cd*s/m^2^) as described previously [23]. The intensity series was divided into ten steps of 0.5 or 1 log units and each trace were averaged from ten responses, with an inter-stimulus interval of 5 s (for < 1 cd*s/ m^2^) or 17 s (for 1 to 25 cd*s/ m^2^). Band-pass filter frequencies were 0.3 and 300 Hz.

#### Steady-state flicker ERG

Dark-adapted flicker ERGs were performed with increasing frequency from 0.5 to 5 Hz at a fixed high mesopic intensity (3 cd*s/m^2^) without background illumination [21]. Responses were averaged either 20 times (for 0.5, 1, 2, and 3 Hz) or 30 times (for 5 Hz) over time, i.e. steady-state recordings. Band-pass filter frequencies were 0.3 and 300 Hz.

#### Flicker ERG with a defined onset

A dark-adapted direct-current amplification (DC) flicker protocol was used at high scotopic conditions (10 mcd*s/m^2^) with traces of 1s length [16] to assess the transition from the resting state towards steady-state conditions. For this analysis, 20 flicker responses were averaged for all frequencies.

This protocol allows to determine the range temporarily not available for light-driven responses due to the elevation of baseline (refractory range, RR), and the remaining dynamic range for light driven responses (DR).

In the DC protocol, the RR is given by the difference between the new flicker baseline at steady state and the baseline before stimulation as the reference. The DR is determined as the new amplitude of the flicker at steady state. Band-pass filter frequency settings for the DC protocol were 0 and 300 Hz.

#### Statistical analysis

The Mann-Whitney rank sum test was used in selected cases to test for statistical significance of differences between ERG amplitudes of *Cnga3*^-/-^ single mutant and *Cnga3*^-/-^ *Hcn1*^-/-^ DKO mice. At a level of p<0.05, differences were considered to be statistically significant and marked with an asterisk (*) in respective figures. Lower p values were further marked with additional asterisks (** for p<0.01, and *** for p<0.001).

### Scanning-Laser Ophthalmoscopy (SLO)

SLO imaging was performed with a HRA 1 system (Heidelberg Engineering, Heidelberg, Germany) in order to visualize the retinal structures of the anesthetized DKO animals (n = 6 eyes) according to previously described procedures [24]. Briefly, the HRA 1 features lasers in the short (visible) wavelength range (488 nm in both and 514 nm in HRA 1 only), and also in the long (infrared) wavelength range (795/830 nm and 785/815 nm). The 488 and 795 nm lasers are used for fluorescein (FLA) and indocyanine green (ICG) angiography, respectively.

### Spectral-Domain Optical Coherence Tomography (SD-OCT)

SD-OCT imaging was performed in DKO mice (n = 6 eyes) in the same session as SLO and it was carried out with a Spectralis HRA+OCT device (Heidelberg Engineering GmbH, Heidelberg, Germany). This device features a super luminescent diode at 870 nm as low coherence light source. Scans are acquired at a speed of 40,000 scans per second and each two-dimensional B-scan contains up to 1536 A-scans. [25]. The images were taken with the equipment set of 30° field of view and with the software Heidelberg Eye Explorer (HEYEX version 5.3.3.0, Heidelberg, Germany).

## Results

### HCN1 Deficiency Leads to an Intensity-Dependent Prolongation of the Rod B-Wave

**Fig 1:**
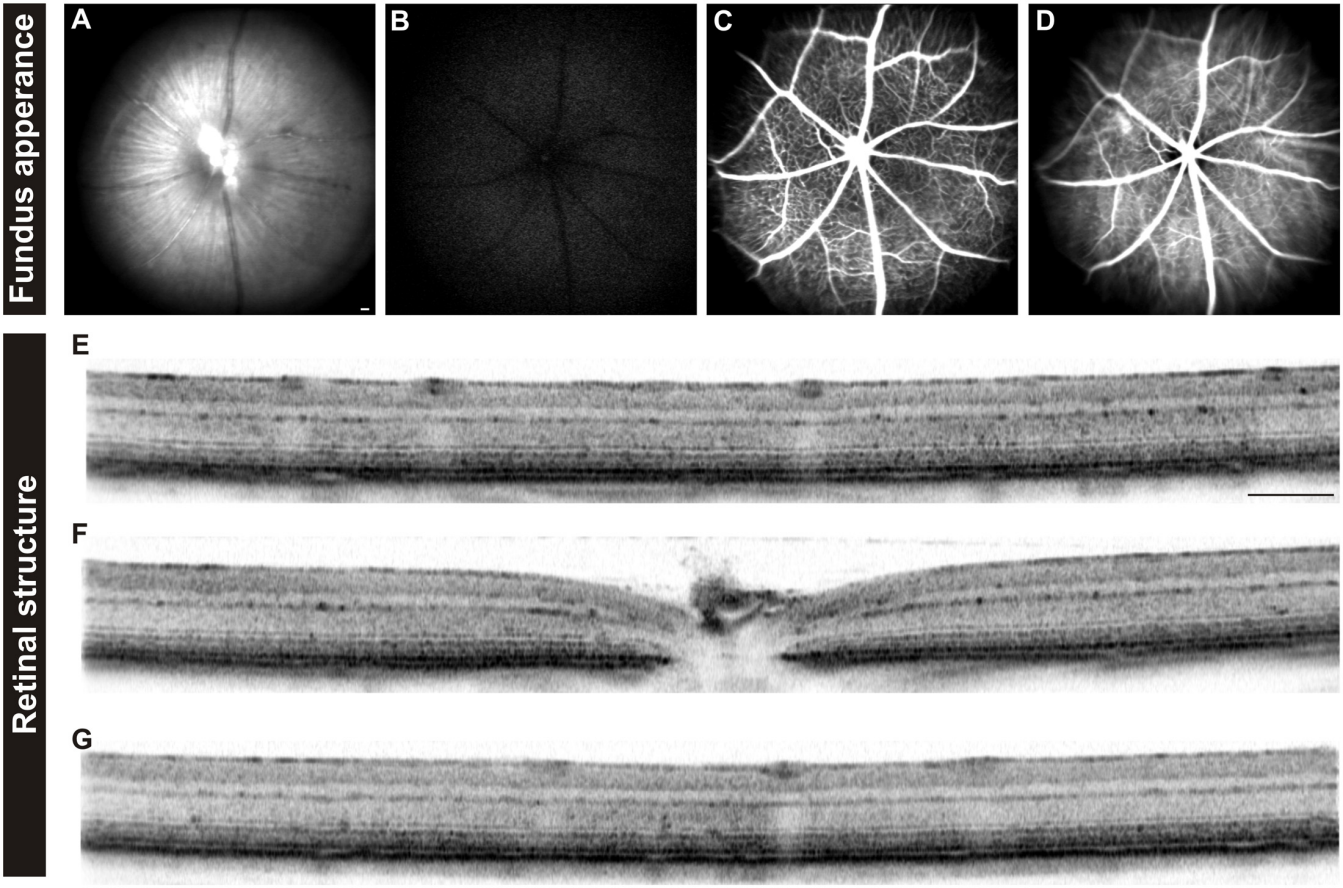
*In vivo* retinal imaging in *Cnga3*^-/-^ *Hcn1*^-/-^ DKO mice. DKO mice (n = 6 eyes) were examined with SLO (A-D) and OCT (E-G). Examinations included fundus native imaging (A), autofluorescence imaging (B), as well as fluorescein angiography (C) and indocyanine green angiography (D). For the OCT analyses, horizontal scans through the dorsal (E), central (F) and ventral part (G) of the retina are shown. *In vivo* imaging of the retina indicates that no signs of retinal degeneration, vascular alterations, or disturbances in the retinal layering of DKO mice. Scale bars (A-G): 200 μm.

Before the functional analysis, we examined whether the lack of HCN1 channels in DKO mice influenced retinal morphology. *In vivo* imaging in DKO mice indicated no abnormalities in retinal structure, neither in SLO fundus images (Fig 1A–1D) nor in OCT cross sections (Fig 1E–1G).

**Fig 2:**
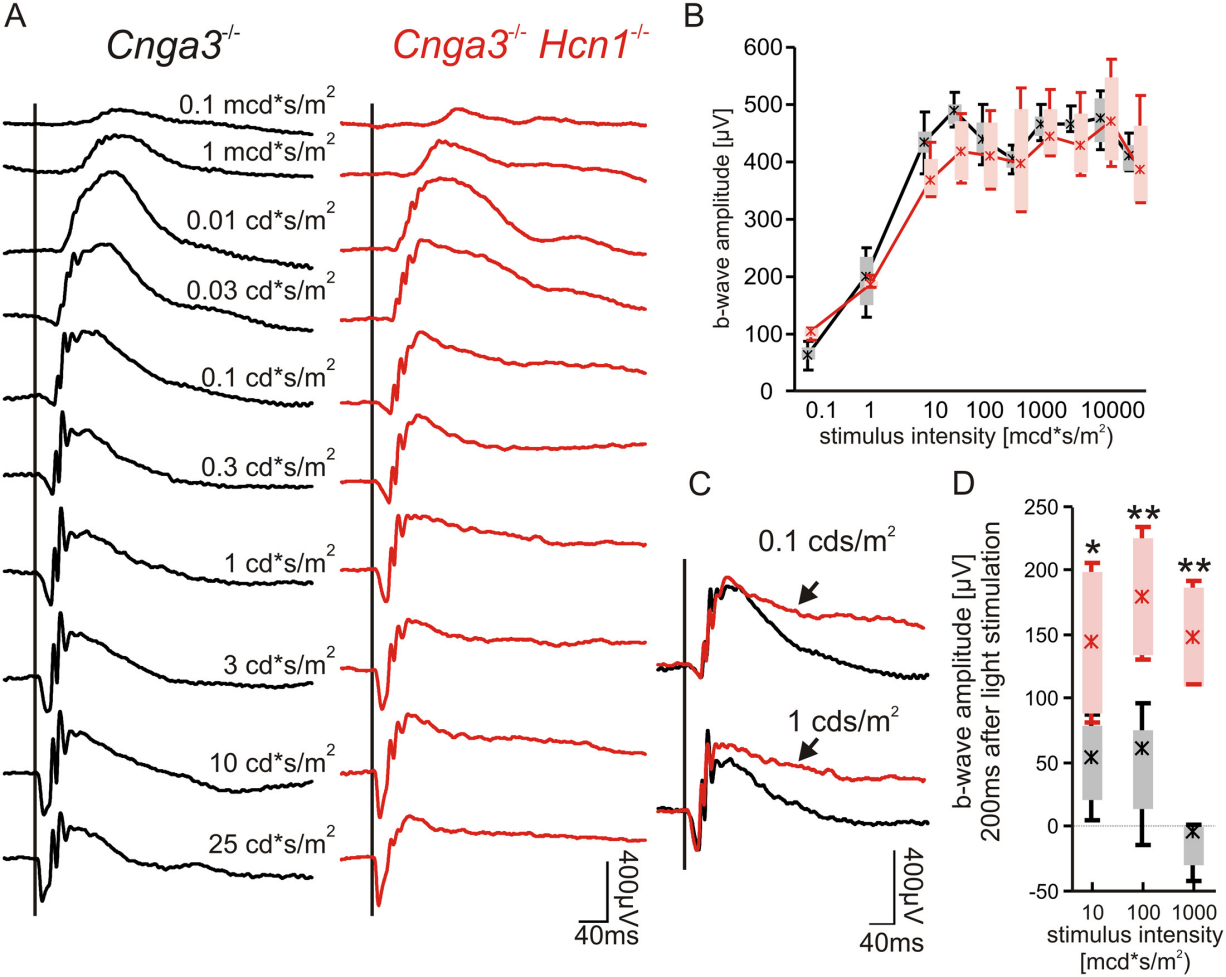
Effect of HCN1 on dark-adapted single flash ERG. (A) Representative ERG traces of an intensity series for a *Cnga3*^-/-^ (left) and a DKO (right) mouse. (B) Quantitative evaluation of b-wave amplitudes (box-and-whisker-plots) for the entire group of *Cnga3*^-/-^ (n = 8 eyes) and DKO (n = 4 eyes). Boxes: 25%-75% quantile range, whiskers: 5% and 95% quantiles, asterisks: median. (C) Superpositions of selected ERG recordings from *Cnga3*^-/-^ mice and DKOs at 0.1 (top) and 1 (bottom) cd*s/m^2^. (D) Amplitudes of selected single flash ERG traces 200 ms after stimulus onset. Statistically significant differences are indicated with asterisks (*p<0.05 for 0.01 cd*s/m^2^ and **p = 0.01 for 0.1 and 1 cd *s/m^2^. Lack of HCN1 leads to an unphysiological prolongation of the b-waves in DKOs at high light intensities.

Next, we asked whether lack of HCN1 channels in this rod-specific DKO line would cause an ERG phenotype similar to that found in non-selective *Hcn1*^-/-^ models [16, 17]. Indeed, the absence of HCN1 channels resulted in unphysiologically prolonged b-wave signals which remained elevated even at 100 ms after light stimulation, corroborating our previous hypotheses. The results are summarized in Fig 2, a compilation of single flash ERGs of *Cnga3*^-/-^ (left) and DKO animals (right) under dark-adapted conditions. For this purpose, we used *Cnga3*^-/-^ mice as control animals since they were found to produce regular rod system response in previous studies involving ERG [19–21].

At low light intensities (Fig 2A; 0.1 and 1 mcd*s/m^2^) ERG data were comparable between both mouse lines; however, a slight tendency towards the HCN1 phenotype was already visible in DKOs at 0.01 cd*s/m^2^ (Fig 2A, right). At brighter light intensities, the prolongation became more pronounced (Fig 2A, right and Fig 2C, arrows). The b-wave amplitudes, determined from the trough to the peak of the positive deflection, were comparable between *Cnga3*^-/-^ and DKO mice (Fig 2B). This is due to the fact that b-wave amplitude measurement does not include later parts of the response waveform, where most of the HCN1 phenotype is present (Fig 2A and 2C). In order to highlight the delayed response termination in DKO, we quantified ERG responses from both mouse lines at a later time-point (200 ms after stimulation) [16]. These data underline that a marked difference is already present at 0.01 cd*s/m^2^ (p = 0.038), but further increases at 0.1 and 1 cd *s/m^2^ (p = 0.010 for both intensities) (Fig 2D).

The intensity series in Fig 2 clearly showed the voltage-dependent activity of HCN1 channels in rod photoreceptor inner segments, since a substantial effect of HCN1-deficiency was discernible only at higher flash intensities above 30 mcd*s/m^2^. In contrast, non-functional HCN1 channels do not affect rod photoreceptor outer segment currents, which was confirmed in *Hcn1*^-/-^ mice [16]; similarly also in DKO mice, no differences were found in the analysis of the a-wave amplitude and implicit-time (data not shown).

In particular, a more detailed comparison between ERG amplitudes of the newly generated DKOs in this work and single *Hcn1*^-/-^ recordings [16, 17] revealed that the prolongation of the b-wave was less pronounced in the DKOs than in *Hcn1*^-/-^ mice, indicating that cone system contributions were indeed present in ERG signals of single mutant mice, whereas these signals were absent in DKOs.

### HCN1*-*Deficiency Reduces the FFF at High Scotopic Conditions

In DKOs, the prolongation of b-waves in the dark-adapted intensity series led us to suppose that, due to the lack of internal HCN1-mediated feedback, rod system responses may be transiently saturated. In ERG, this would become noticeable as a reduced ability to respond to repetitive stimuli, i.e. a lowered flicker fusion frequency (FFF). To investigate this hypothesis, we assessed the transition from the resting state to steady-state conditions by recording single traces of flicker ERG with a defined onset. This enabled us to retain information about the signal baseline before and during light stimulation, including the refractory range (RR) and the remaining dynamic range (DR).

**Fig 3:**
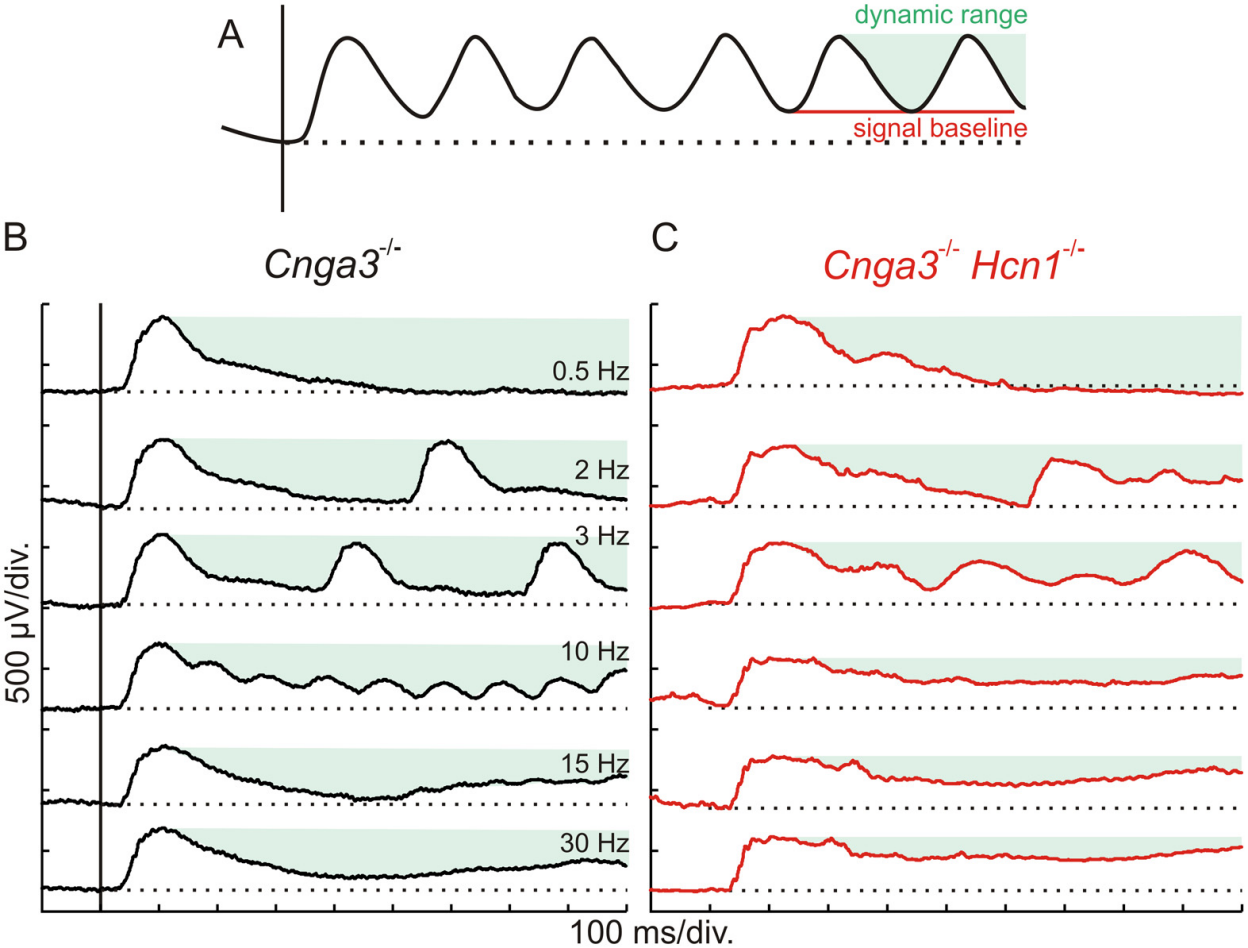
Effect of HCN1 on flicker fusion frequency under high scotopic conditions. (A) Scheme of an ERG flicker response, highlighting characteristic parameters of the record: the dynamic range (DR, green shaded area), the signal baseline (red line) and the baseline prior to the stimulus (dotted black line). (B, C) Scotopic direct current ERGs of a *Cnga3*^-/-^ and a DKO mouse under high scotopic conditions (0.01 cd*s /m^2^). In *Cnga3*^-/-^ controls (B), increasing inability of the rod system response to reach signal baseline decreases the DR, leading to a flicker fusion frequency (FFF) of about 15Hz. The additional prolongation of the rod response due to HCN1 deficiency limits its DR to a much greater extent and thus reduces the FFF (C). In total, DC measurements were performed in n = 6 eyes for Cnga3^-/-^ mice and n = 4 eyes for DKO mice.

Two key factors that influence the flicker ERG are the signal baseline level and the basal DR, which are illustrated in Fig 3A. In a frequency series, signal baseline level (Fig 3A, red line) is increasingly elevated whenever single ERG responses are unable to reach the initial level before onset of the following response (dotted line), creating a refractory range (RR) spanned by the original baseline (dotted line) and the new baseline level. An increase in RR does in turn reduce the DR (green shaded area) for subsequent responses.

The flicker response characteristics of *Cnga3*^-/-^ and DKOs are presented in Fig 3B and 3C. In *Cnga3*^-/-^ animals, the rod system was able to follow high flicker frequencies up to about 12–18 Hz. In the representative flicker series shown here, the FFF was slightly above 15Hz and traces at frequencies above FFF were close to single flash-like responses. In contrast, the prolongation of the rod system response due the lack of HCN1 channels caused a remarkable reduction of the FFF in DKOs down to 7-10Hz, together with an elevation of the baseline abnormally increasing with frequency (Fig 3C). At frequencies above the FFF, the flicker ERG signal remained elevated with no remaining DR, resulting in a step-like response as described for *Hcn1*^-/-^ in our previous study [17]. These data corroborate the important role of HCN1 channels in rod vision. In particular, HCN1 channels enable the rod system to resolve higher frequencies by extending their DR at the photoreceptor level at intensities of 0.01 cd*s/m^2^ and above.

### HCN1*-*Deficiency Reduces the FFF at High Mesopic Conditions

**Fig 4:**
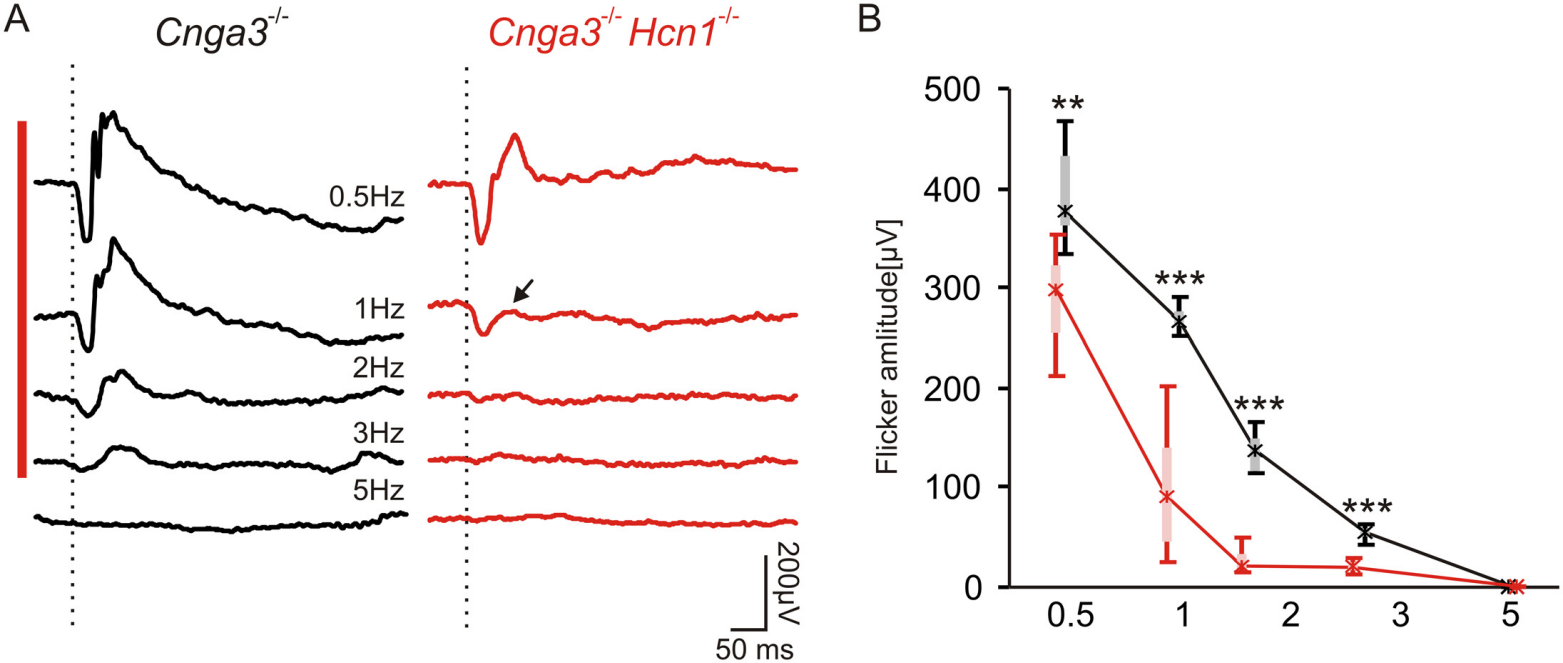
Rod flicker ERG under high mesopic conditions. (A) Steady-state flicker ERG of a *Cnga3*^-/-^ (left) and a DKO (right) mouse under high mesopic conditions (3 cd*s/m^2^). (B) Quantification of flicker ERG amplitudes of *Cnga3*^-/-^ (n = 8 eyes) and DKOs (n = 8 eyes), indicating a strong reduction of rod flicker responses in DKO mice. Boxes: 25%-75% quantile range, whiskers: 5% and 95% quantiles, asterisks: median. Statistically significant differences are indicated with asterisks (**p<0.01 for 0.5 Hz and ***p<0.001 for 1–3 Hz). HCN1 deficiency reduces the ability to follow flicker under high mesopic conditions even further (A, red bar).

Finally, we addressed the question whether the lack of HCN1 influences the FFF also under high mesopic conditions at 3 cd*s/m². We used conventional steady-state ERG flicker recordings, where responses were continuously averaged. The normal rod system is able to respond under these conditions only to low temporal frequencies of up to 3 Hz (Fig 4A, red bar) [21]. In DKO, ERG responses were even more reduced with increasing frequency (Fig 4A, right), with a tendency to a stronger reduction of the positive deflection relative to the negative deflection (Fig 4A right, arrow). A summary of flicker amplitude data from *Cnga3*^-/-^ and DKO mice, including the analysis of the positive deflection (Fig 4B), further illustrates the significant reduction in FFF at 0.5 Hz (p = 0.002) and above (p<0.001 for 1–3 Hz).

## Discussion

In this study, we investigated the role of HCN1 channels in rod photoreceptors for the enhancement of rod system responsivity under conditions of light exposure. To isolate HCN1 channel actions in rod system responses, we generated *Hcn1*^-/-^ *Cnga3*^-/-^ double knockout mice. The *Cnga3*^-/-^ line is a model of achromatopsia type 2 and particularly specific in the removal of any light-activated cone signalling, while the rod system stays functionally intact [18]. *Hcn1*^-/-^ *Cnga3*^-/-^ double knockout mice thus allow to study rod-driven visual activity in HCN1-deficient mutants. We found that a lack of HCN1-mediated feedback in rod photoreceptor cells indeed prolongs rod responses and saturates the downstream retinal network during bright light stimulation (Fig 2). In ERG analyses, this was noticeable as a reduced dynamic range (DR) and a lowered flicker fusion frequency (FFF, Figs 3 and 4). Feedback mechanisms are common strategies of the mammalian retina to avoid saturation of the neuronal network. A further example is the triad of synapses between rod bipolar cells and AII and A17 amacrine cells. These triads modulate rod bipolar output signals and consequently influence the saturation of rod vision [23]. When comparing these adaptive mechanisms, they appear to share the same goal: To avoid saturation without compromising sensitivity. Evolutionary, one may speculate that the maximal sensitivity of vision in darkness is an advantage that is equally helpful to the ability to rapidly perceive light-evoked signals again following a saturating stimulus, both for predators and prey.

With more and more details about the functional components of photoreceptors emerging, modelling of entire cells comes within reach. Our rod-specific *in vivo* data does provide first-hand information for the implementation of such models and generally for computational studies dealing with saturation of the rod system.

In summary, we show that HCN1 channel feedback enhances the dynamic range of the rod system in order to limit the period of saturation following bright light exposure, while leaving rod sensitivity untouched. The newly generated *Cnga3*^-/-^ *Hcn1*^-/-^ double mutant is a valuable specific model and a crucial building block to understand and model the role of HCN1 channels in shaping mammalian vision.

## References

[1] Sharpe LT, Stockman A. Rod pathways: the importance of seeing nothing. Trends Neurosci. 1999; 22:497–504 10529817 10.1016/s0166-2236(99)01458-7

[2] Wässle H. Parallel processing in the mammalian retina. Nat Rev. Neurosci. 2004; 5:747–757 15378035 10.1038/nrn1497

[3] Rieke F, Rudd ME. The challenges natural images pose for visual adaptation. Neuron. 2009; 64:605–16. 10.1016/j.neuron.2009.11.028 20005818

[4] Völgyi B, Deans MR, Paul DL, Bloomfield SA et al Convergence and segregation of the multiple rod pathways in mammalian retina. J Neurosci. 2004; 24:11182–92 15590935 10.1523/JNEUROSCI.3096-04.2004 PMC2834589

[5] Kolb H. The organization of the outer plexiform layer in the retina of the cat: electron microscopy observations. J Neurocytol. 1977; 6:131–153. 856949 10.1007/BF01261502

[6] Abd-El-Barr MM, Pennesi ME, Saszik SM, Barrow AJ, Lem J, Bramblett DE et al Genetic dissection of rod and cone pathways in the dark-adapted mouse retina. J Neurophysiol. 2009; 102:1945–55. 10.1152/jn.00142.2009 19587322 PMC2746771

[7] Lee EJ, Han JW, Kim HJ, Kim IB, Lee MY, Oh SJ et al The immuncyto-chemical localization of connexin 36 at rod and cone gap junctions in the guinea pig retina. Eur J Neurosci. 2003; 18:2925–2934. 14656288 10.1046/j.1460-9568.2003.03049.x

[8] Feigenspan A, Janssen-Bienhold U, Hormuzdi S, Monyer H, Degen J, Söhl Gl et al Expression of connexin36 in cone pedicles and OFF-cone bipolar cells of the mouse retina. J Neurosci. 2004; 24:3325–3334. 15056712 10.1523/JNEUROSCI.5598-03.2004 PMC6730041

[9] Raviola E, Gilula NB. Gap junctions between photoreceptor cells in the vertebrate retina. Proc Natl Acad Sci. USA. 1973; 70:1677–1681. 4198274 10.1073/pnas.70.6.1677 PMC433571

[10] Nelson R. Cat cones have rod input: a comparison of the response properties of cones and horizontal cell bodies in the retina of the cat. J Comp Neurol. 1977; 172:109–135. 838876 10.1002/cne.901720106

[11] Müller F, Scholten A, Ivanova E, Haverkamp S, Kremmer E, Kaupp UB. HCN channels are expressed differentially in retinal bipolar cells and concentrated at synaptic terminals. Eur J Neurosci. 2003; 17:2084–96. 12786975 10.1046/j.1460-9568.2003.02634.x

[12] Ivanova E, Müller F. Retinal bipolar cell types differ in their inventory of ion channels. Vis Neurosci. 2006; 23:143–54. 16638168 10.1017/S0952523806232048

[13] Della Santina L, Piano I, Cangiano L, Caputo A, Ludwig A, Cervetto L et al Processing of retinal signals in normal and HCN deficient mice. PLOS ONE. 2012; 7:e29812 10.1371/journal.pone.0029812 22279546 PMC3261154

[14] Barrow AJ, Wu SM. Low-conductance HCN1 ion channels augment the frequency response of rod and cone photoreceptors. J Neurosci 2009; 29:5841–5853. 10.1523/JNEUROSCI.5746-08.2009 19420251 PMC2695939

[15] Knop GC, Seeliger MW, Thiel F, Mataruga A, Kaupp UB, Friedburg C et al Light responses in the mouse retina are prolonged upon targeted deletion of the HCN1 channel gene. Eur J Neurosci. 2008; 28:2221–2230. 10.1111/j.1460-9568.2008.06512.x 19019198

[16] Seeliger MW, Brombas A, Weiler R, Humphries P, Knop G, Tanimoto N. Modulation of rod photoreceptor output by HCN1 channels is essential for regular mesopic cone vision. Nat Commun. 2011; 2:532 10.1038/ncomms1540 22068599

[17] Nolan MF, Malleret G, Lee KH, Gibbs E, Dudman JT, Santoro B et al The hyper-polarization-activated HCN1 channel is important for motor learning and neuronal integration by cerebellar Purkinje cells. Cell. 2003; 115:551–64. 14651847 10.1016/s0092-8674(03)00884-5

[18] Biel M, Seeliger MW, Pfeifer A, Kohler K, Gerstner A, Ludwig A et al Selective loss of cone function in mice lacking the cyclic nucleotide-gated channel CNG3. Proc Natl Acad Sci U S A. 1999; 96:7553–7. 10377453 10.1073/pnas.96.13.7553 PMC22124

[19] Tanimoto N, Muehlfriedel RL, Fischer MD, Fahl E, Humphries P, Biel M et al Vision tests in the mouse: Functional phenotyping with electroretinography. Front Biosci (Landmark Ed). 2009; 14:2730–7. 19273231 10.2741/3409

[20] Tanimoto N, Sothilingam V, Seeliger MW. Functional phenotyping of mouse models with ERG. Methods Mol Biol. 2013; 935:69–78. 10.1007/978-1-62703-080-9_4 23150360

[21] Tanimoto N, Sothilingam V, Kondo M, Biel M, Humphries P, Seeliger MW. Electroretinographic assessment of rod- and cone-mediated bipolar cell pathways using flicker stimuli in mice. Sci Rep. 2015; 5:10731 10.1038/srep10731 26029863 PMC5377071

[22] Haverkamp S, Michalakis S, Claes E, Seeliger MW, Humphries P, Biel M et al Synaptic plasticity in CNGA3(-/-) mice: cone bipolar cells react on the missing cone input and form ectopic synapses with rods. J Neurosci. 2006; 26:5248–55. 16687517 10.1523/JNEUROSCI.4483-05.2006 PMC6674253

[23] Tanimoto N, Sothilingam V, Euler T, Ruth P, Seeliger MW, Schubert T. BK channels mediate pathway-specific modulation of visual signals in the in vivo mouse retina. J Neurosci. 2012; 32:4861–6 10.1523/JNEUROSCI.4654-11.2012 22492042 PMC6620922

[24] Seeliger MW, Beck SC, Pereyra-Munoz N, Dangel S, Tsai JY, Luhmann UF et al In vivo confocal imaging of the retina in animal models using scanning laser ophthalmoscopy. Vision Res. 2005; 45: 3512–9. 16188288 10.1016/j.visres.2005.08.014

[25] Fischer MD, Huber G, Beck SC, Tanimoto N, Muehlfriedel R, Fahl E et al Noninvasive, in vivo assessment of mouse retinal structure using optical coherence tomography. PLOS ONE. 2009; 4:e7507 10.1371/journal.pone.0007507 19838301 PMC2759518

